# Melatonin pretreatment on exosomes: Heterogeneity, therapeutic effects, and usage

**DOI:** 10.3389/fimmu.2022.933736

**Published:** 2022-09-16

**Authors:** Zilan Zhou, Ruiping Wang, Jie Wang, Yujia Hao, Qingpeng Xie, Lu Wang, Xing Wang

**Affiliations:** ^1^School and Hospital of Stomatology, Shanxi Medical University, Taiyuan, China; ^2^Shanxi Province Key Laboratory of Oral Diseases Prevention and New Materials, Taiyuan, China; ^3^Science and Technology Information and Strategy Research Center of Shanxi, Taiyuan, China

**Keywords:** exosomes, melatonin, pretreatment, heterogeneity, therapeutic

## Abstract

The therapeutic outcomes of exosome-based therapies have greatly exceeded initial expectations in many clinically intractable diseases due to the safety, low toxicity, and immunogenicity of exosomes, but the production of the exosomes is a bottleneck for wide use. To increase the yield of the exosomes, various solutions have been tried, such as hypoxia, extracellular acidic pH, etc. With a limited number of cells or exosomes, an alternative approach has been developed to improve the efficacy of exosomes through cell pretreatment recently. Melatonin is synthesized from tryptophan and secreted in the pineal gland, presenting a protective effect in pathological conditions. As a new pretreatment method, melatonin can effectively enhance the antioxidant, anti-inflammatory, and anti-apoptotic function of exosomes in chronic kidney disease, diabetic wound healing, and ischemia-reperfusion treatments. However, the current use of melatonin pretreatment varies widely. Here, we discuss the effects of melatonin pretreatment on the heterogeneity of exosomes based on the role of melatonin and further speculate on the possible mechanisms. Finally, the therapeutic use of exosomes and the usage of melatonin pretreatment are described.

## Introduction

The therapeutic outcomes of exosome-based therapies have greatly exceeded initial expectations in clinical refractory diseases (1), such as cardiovascular diseases, neurodegenerative diseases, and cancer progression (2). Compared with cell therapy, the exosome therapeutic platform has the advantages of safety, low toxicity, and immunogenicity (3), which can be administered repeatedly (4). As a new therapeutic platform, there are still many technical problems during application. The low yield of exosomes remains a bottleneck in the large-scale implementation (5).

Isolation of exosomes is the first step. Standard exosome isolation techniques are based on differential ultracentrifugation, size, bead, and polymer (6). All these methods are considered tedious and challenging because they require large sample volumes, multi-step operations, and several days. The low production of exosomes greatly hinders their wide application (5, 7). In the clinic, a dose of 10 (8) exosomes is typically needed per treatment for a patient (9), which requires more than a week and several hundred milliliters of cell supernatant (10, 11). Thus, current productivity is insufficient (12).

Various solutions have been tried to increase the production of exosomes. For example, Ban et al. found that exosome production was increased through changes in environmental pH values (13), but pH affected the stability of the exosomes. Wang et al. reported that toward microfluidic-based exosome isolation, it affects the biological functions of exosomes, such as the targeting of exosomes (6). Hao et al. suggested that knockdown or overexpression of certain genes promotes the release of exosomes but destroys the natural structure of exosomes (8). Up to now, how to regulate exosomes release without impacting the native structure or functions remains unsolved (14).

With a limited number of cells or exosomes, an alternative approach has been developed to improve the efficacy of exosomes through cell pretreatment recently. Melatonin (*N*-acetyl-5-methoxytryptamine) can effectively enhance the antioxidant, anti-inflammatory, and anti-apoptotic functions of exosomes. Exosomes extracted from cells pretreated with Melatonin are named MT-exosomes (15, 16). Some studies revealed that the therapeutic potency of exosomes can be improved by melatonin pretreatment in chronic kidney disease (CKD), ischemia-reperfusion (IR) treatments, and wound healing in diabetes (15–18). It is considered a booster to break through the barrier of exosome application quickly. However, there is great variability in the effect on MT-exosomes and how to use the melatonin pretreatment.

Here, we discuss the effects of melatonin pretreatment on the heterogeneity of exosomes based on the role of melatonin. Given the application of melatonin pretreatment on exosomes is immature, we further speculate on the possible mechanisms. Finally, the therapeutic use of exosomes and the usage of melatonin pretreatment are summarized. Encouragingly, melatonin pretreatment provides a unique and new approach to the exosomes, which gives a new way to overcome the “low yield bottlenecks” of exosomes in the therapeutic platform.

## Melatonin and melatonin pretreatment

**Figure 1:**
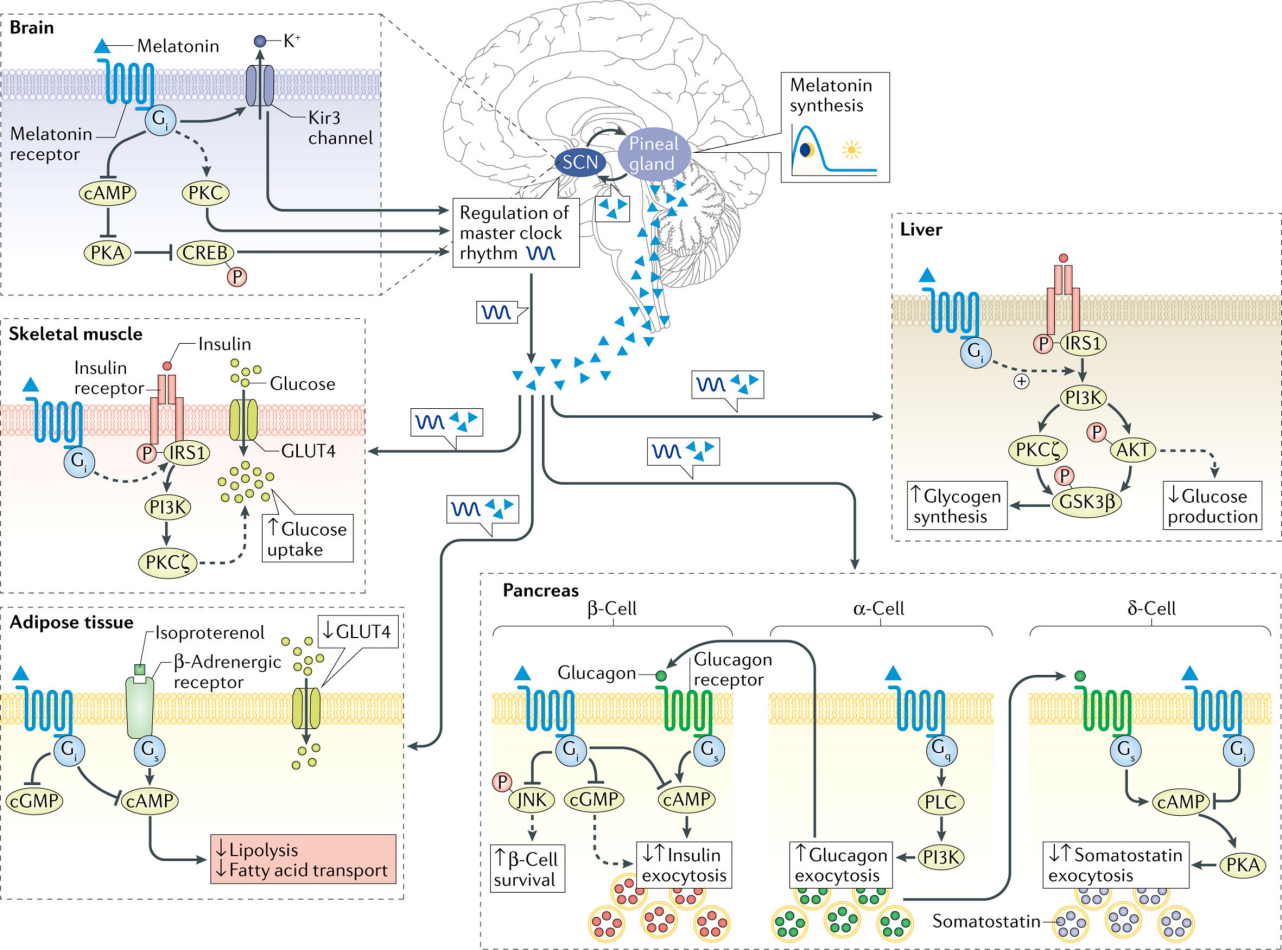
Metabolic processes influenced by melatonin signaling in peripheral and central tissues. Broken lines correspond to the function of melatonin, which exact pathways remain to be elucidated. The dark blue wave-formed line illustrates the circadian clock oscillation. Reprinted by permission from Copyright clearance center: Springer Nature, Nature Reviews Endocrinology, Melatonin in type 2 diabetes mellitus and obesity, Angeliki Karamitri et al, COPYRIGHT (2018).

Melatonin, an indole derivative discovered in 1958, is a natural neurotransmitter (19). Controlled by the master clock of the suprachiasmatic nucleus (SCN) of the hypothalamus, melatonin is secreted by the pineal gland according to the circadian rhythm (20) (**Figure 1**). Melatonin has been known for decades as an animal hormone. However, later in the 1990s, it was also found in plants and is now widely considered a common parental molecule that permeates and influences the viability of all cells (21).

Melatonin may be a cornucopia, with various therapeutic effects such as antioxidant, anti-inflammatory, anti-infective, and anti-tumor, assisting to treat fertility disorders, osteoporosis, cardiovascular disease, Alzheimer’s disease, obesity, influenza, gastrointestinal tumors, and non–small cell lung cancer (22, 23). In many countries in Europe and the United States, melatonin is widely used both as a prescription drug and as a non-prescription supplement (24).

Melatonin can also be used for pretreatment, meaning that it is added in advance of treatment or injury. Melatonin pretreatment not only improves the therapeutic effect of some diseases (25–27), such as blast injury and kidney IR injury, but also alleviates the toxicity of drugs (28) and plants (**Table 1**) (29–33). Studies have found that melatonin pretreatment improves the survival rate and angiogenesis of cells (34), as well as the therapeutic effect on solid organ cells (35). Pan et al. found that melatonin inhibited the increase in the ameloblast-lineage cell line (ALC) number in a time-dependent and dose-dependent manner (0, 10^–10^, 10^–8^, 10^–6^, 10^–4^, and 10^−3^ M). With the increase in melatonin concentration, the alkaline phosphatase activity of ALCs gradually increased (36). Cells from different sources respond differently to melatonin concentrations. Cucielo et al. found that melatonin alters the mitochondrial membrane and the size of human ovarian carcinoma cells (SKOV-3 cells). After melatonin treatment, SKOV-3 cells showed a statistically significant reduction in mitochondrial metabolism at all concentrations (1.6, 3.2, and 4 mM) (37). Wang et al. found that P21 (cell cycle–associated gene) expression was decreased in granulosa cells treated with the high-concentration (10^−5^ M) melatonin and increased in that treated with the low-concentration (10^−9^ M) melatonin (38). Moreover, melatonin pretreatment also affects the exosome release from the donor cells and significantly enhances the therapeutic effects of MT-exosomes (39, 40), which are discussed in the following sections.

**Table 1:** Related research on melatonin pretreatment.

Author (Year)	Subjects	Methods	Concentration	Results
de Farias et al. (2022) (25)	Zebrafish	Melatonin was injected directly into the aquarium 3 nights, and 7 nights before inducing seizures	100 nM	Melatonin promotes a neuroprotective response against the epileptic profile in zebrafish.
Liang et al. (2021) (28)	Neonatal C57BL/6J mice	Mice were treated with melatonin at 0.5 h before sevoflurane anesthesia.	10 mg/kg	Melatonin pretreatment alleviates the long-term synaptic toxicity and dysmyelination induced by neonatal sevoflurane exposure.
Zhang et al. (2021) (26)	8-week-old male C57BL/6 mice	Mice were intraperitoneally injected with melatonin for 7 consecutive days before blast injury	20 mg/kg	Melatonin pretreatment alleviated blast-induced behavioral abnormalities in mice.
Jahan et al. (2021) (29)	Tomato seedlings (the fourth leaf stage)	The seedlings were foliar-sprayed with melatonin continued for 7 days before high-temperature stress	100 µM	Melatonin treatment markedly attenuated heat-induced leaf senescence.
Tousi et al. (2020) (30)	Mallow plant seeds	The plants were placed in the pots with melatonin. After 2 days, and Cd (NO_3_)_2_.4H_2_O was added.	0, 15, 50, and 100 µM	Melatonin could reduce oxidative stress and improve biomass in the plants exposed to cadmium.
Yang et al. (2020) (14)	8–9-week-old male C57BL/6 mice	Melatonin was intraperitoneally administered 24 h and 1 h before renal ischemia-reperfusion injury	20 mg/kg	Melatonin treatment provides protection for the kidney against ischemia-reperfusion injury by enhancing autophagy.
De Butte et al. (2020) (31)	Female Sprague–Dawley rats	Melatonin pellets (subcutaneous implants) were present for 2 weeks prior to bilateral common carotid occlusion.	5 mg (60-day time)	Melatonin retains the ability to protect hippocampal neurons from ischemia-induced damage in older female rats.
Wang et al. (2019) (32)	Male ICR mice	Mice received melatonin 15 min prior to methamphetamine administration	2.5, 5, and 10 mg/kg	Melatonin has the capacity to reverse methamphetamine-induced aggressive behaviors.
Nawaz et al. (2018) (33)	Watermelon seedlings	Watermelon seedlings were pretreated with melatonin	0.1 μM	Melatonin could be utilized to reduce the availability of vanadium to plants, and improve plant growth and vanadium stress tolerance.

## Effects of melatonin pretreatment on exosome heterogeneity

**Figure 2:**
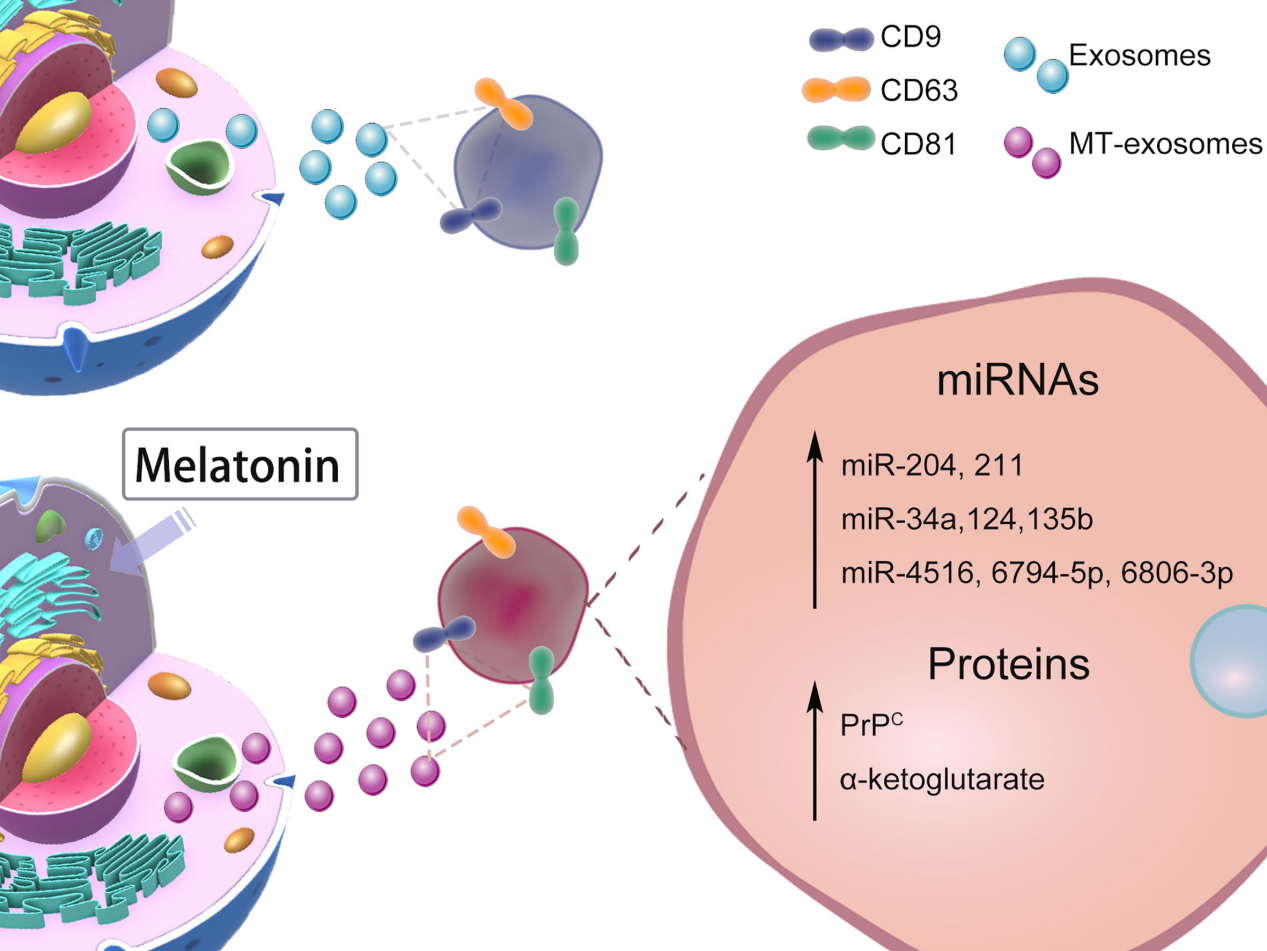
Effects of Melatonin Pretreatment on Exosome heterogeneity: size, production, miRNAs, proteins. The heterogeneity of the effect of melatonin pretreatment on exosomes is still controversial. We mainly list the changes in size, production, and content of exosomes reported by current related studies.

The heterogeneity of exosomes mainly includes the size and content of exosomes (41), functional effects on recipient cells, and cell origin; different combinations lead to complex heterogeneity of exosomes (2). Whether melatonin pretreatment affects exosome heterogeneity or not is controversial. In this section, we mainly discuss the heterogeneity of MT-exosomes including size, production, microRNA (miRNA), and protein (**Figure 2**). The effects of melatonin pretreatment on exosomes, among others, may vary with specific cell types and conditions.

### Size and production of MT-exosomes

Melatonin pretreatment affects the exosome size and production with variable effects. On the one hand, some studies suggest that melatonin pretreatment has no significant effect on the size and production of exosomes. Liu et al. observed that both MT-exosomes and exosomes were oval bilayer lipid membrane vesicles with a diameter of about 120 nm and had no significant difference in production (approximately 7.0 × 10^8^ and 7.5 × 10^8^, respectively) (15). Cheng et al. also demonstrated that melatonin pretreatment does not affect exosome production in hepatocellular carcinoma (HCC) (42).

On the other hand, some researchers hold different views (43). Abd-Elhafeez et al. found melatonin pretreatment significantly increases the size and production of exosomes in telocytes (44). Pournaghi et al. reported that melatonin pretreatment significantly reduced the exosome size but increased the production in bovine granulosa cells (45). Ozansoy et al. found that the production of exosomes in SH-SY5Y human neuroblastoma cells decreased by 36.23% after pretreatment with melatonin (46).

Given the current evidence, melatonin may affect the exosomes from various donor cells through different biogenesis mechanisms (45). Heterogeneity can endow unique properties on exosomes based on the organ and tissue from which they originate (2). In addition, considering the difference in experimental conditions, we believe that the concentration of melatonin is another significant factor, which is discussed in the following section.

### miRNAs change in MT-exosomes

The heterogeneity of exosomes includes size and yield, as well as content (miRNAs and proteins). In CKD-MSCs, Yoon et al. found that miR-4516, miR-6806-3p, and miR-6794-5p were increased in MT-exosomes (16). In smooth muscle, Xu et al. reported that melatonin pretreatment significantly increased the expression of miR-204 and miR-211 in exosomes from smooth muscle cells (47). In bone, it is considered that melatonin pretreatment increases the content of miR-181 in exosomes and further improves the osteogenesis effect of exosomes (48).

In chronic inflammatory diseases or autoimmune diseases, Heo et al. found the specific miR-34a, miR-124, and miR-135b of anti-inflammatory macrophages (M2) expressed significantly higher in MT-exosomes than in exosomes (49). MT-exosomes can additively attenuate inflammation, which may provide a therapeutic target for immunoregulation and anti-inflammatory responses (50).

### Proteins change in MT-exosomes

Specific molecules on the surface of exosomes determine the internalization, immune escape, and targeting of the delivery of exosomes (51), which mediate signaling and help them escape the internalization and into target cells (52). Heo et al. suggested that the exosome marker CD9 was not affected by melatonin pretreatment (49). The same results were found in the CD9, CD81, CD63, apoptosis-linked gene 2-interacting protein X (Alix), and tumor susceptibility gene 101 (TSG101) (2), which demonstrates that exosomes and MT-exosomes have similar surface proteins (15, 16, 42, 49).

After melatonin pretreatment, the internal proteins carried by exosomes change differently. In the murine hindlimb ischemia model, the expression of the cellular prion protein (PrP^C^) significantly increases in MT-exosomes of healthy MSCs and CKD-MSCs (16), enhancing the proliferation and release of angiogenic cytokines. Liu et al. found that the α-ketoglutarate (metabolite contents) level from MT-exosomes was increased to alleviate inflammatory response (53).

Interestingly, Ozansoy et al. found that the secretion of tau in exosomes of the amyloid-beta (Aβ) toxicity model varied with the time sequence of melatonin treatment. The amount of total tau was not impacted by melatonin pretreatment but significantly reduced by post-treatment of melatonin in exosomes (46). They suggested that the amyloid-beta and melatonin play an important role in the mechanism of exosomes, and the effect of exosomal tau content was time-sensitive in the 8-hour application cycle.

As the product of cells, exosomes are heterogeneous, and it is difficult to isolate and to pool identical exosomes in exosome studies. Currently, there are many methods for analyzing exosome heterogeneity, such as flow cytometer, immunoaffinity capture, and asymmetric flow-field flow fractionation (54). Super-resolution microscopy enables us to accurately quantify multiple characteristics of exosome secretion by single human macrophages (55). This may be a crucial way to resolve and understand the heterogeneity of MT-exosomes.

## Possible mechanism of melatonin pretreatment on exosomes

**Figure 3:**
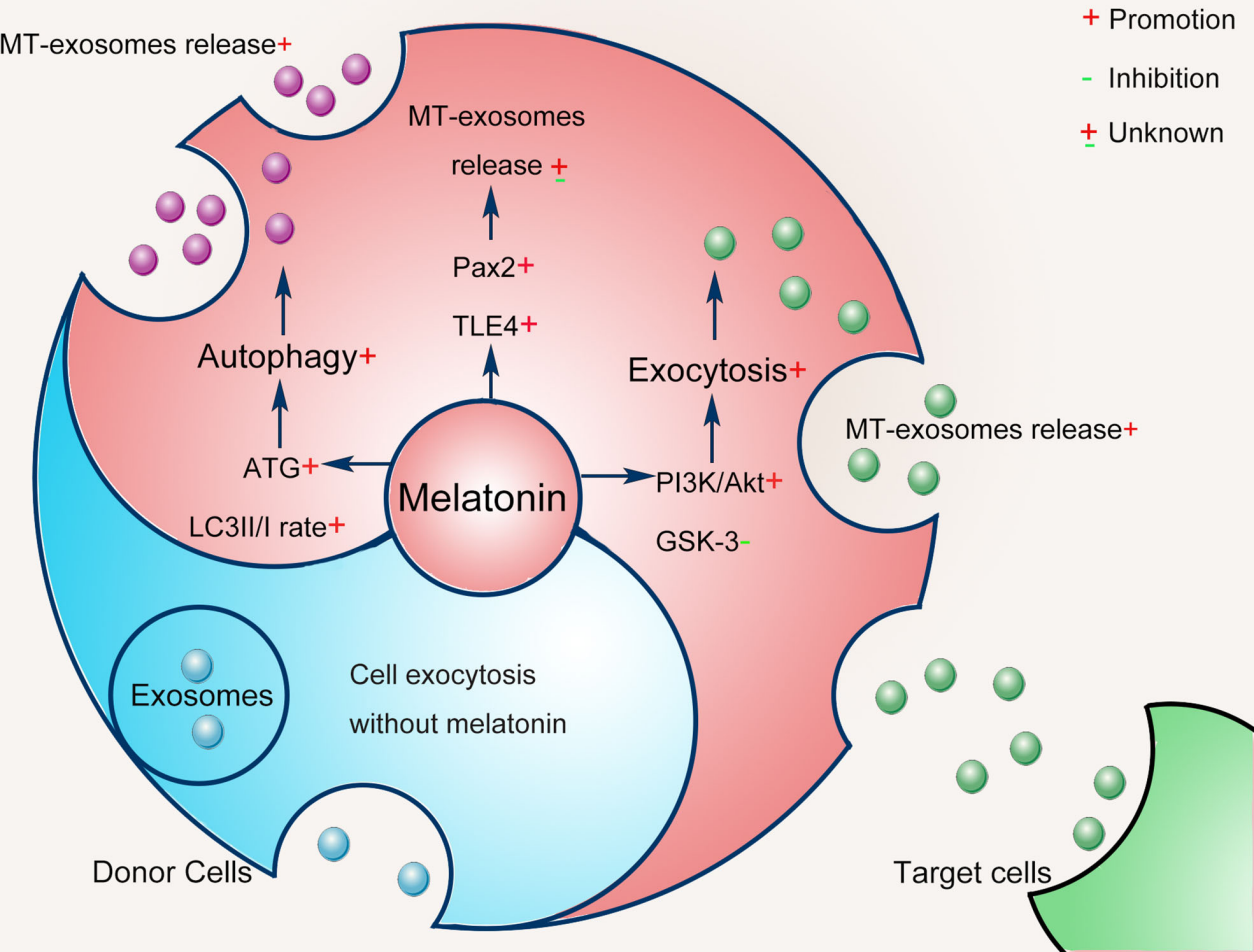
Possible mechanisms of melatonin pretreatment on exosomes: cell exocytosis and autophagy.

It is inconclusive that the heterogeneity of exosomes is more or less affected by melatonin pretreatment. Alterations in exosome heterogeneity may contribute to the complexity of exosomes, the mechanism of which remains unknown. In this section, we further speculate on the mechanism by which exosomes are pretreated with melatonin as follows (**Figure 3**). Notably, the mechanisms involved may vary with specific cell types and different conditions.

Generally, exosomes are produced through the endocytic pathway and released through exocytosis (41). Exosomes originate from the terminal endosomes formed by the inward budding of the multivesicular bodies (MVBs) membrane (56, 57). After MVBs dock with the plasma membrane, exosomes are released to the extracellular through exocytosis (58), and midbody remnants are produced by cytokine abscission of interconnecting bridges between dividing cells (59). Otherwise, a part of the MVBs is transported to be merged with the lysosomes to degrade the cargo, and the autophagosome encapsulating the protein and/or organelles can fuse with the endosomes forming an amphisome, which then undergoes lysosomal breakdown (60). Exosomes are regulated in different steps, including their biogenesis, cargo selection, and cell-specific uptake. Among them, exosome biogenesis is a multi-step biological process, which represents the complexity of exosome composition and is also the focus of exosome-related research (61).

Melatonin pretreatment regulates the biogenesis of exosomes by modulating the exocytosis of donor cells. Melatonin pretreatment enhances cell exocytosis to release exosomes *via* the phosphatidylinositol 3-kinase/protein kinase B (PI3K/Akt) axis inhibiting the activity of glycogen synthase kinase 3 (GSK-3) (62). Melatonin pretreatment also increases the flexibility and fluidity of donor cell membranes and promotes cell exocytosis (63). Moreover, melatonin on fatty acid metabolism may be a molecular mechanism affecting the production of exosomes from donor cells (43, 45).

Melatonin pretreatment affects the biogenesis of exosomes by triggering cell exocytosis and autophagy (64, 65). Autophagy is a regulated self-degrading process that modulates changes in exosome biogenesis in response to changes in external stimuli (66). The melatonin pretreatment in the donor cell causes cell autophagy, which likely changes the biogenesis of exosomes (67). Different autophagy proteins have been proven to regulate exosome biogenesis (68, 69). Autophagy-related proteins (ATGs) are essential regulators of cytosol and membrane autophagy (70). Guo et al. found that ATG5 and ATG16L1 have common signaling pathways between autophagy and exosome biogenesis (69). Melatonin directly stimulates autophagy by activating the ATG4, 5, 7, 10, 12, and 16, increasing the microtubule-associated protein 1A/1B light chain 3 (LC3) II/I rate (71). Melatonin can enhance the autophagy mechanism of cells (67), increasing MVB autophagosome fusion and generating amphisomes (72). Then, amphisomes use specific GTPases (including Rab8a and Rab27a) to release exosomes and autophagic contents (68). Thus, the exosome production and the contents they carry are altered.

To further explore the possible effects of melatonin pretreatment on exosome biogenesis, Amini et al. conducted a bioinformatics analysis based on common genes associated with the melatonin signaling pathway, exosomes biogenesis, and the Wnt cascade (43). Results showed that Pax2 and the transducin-like enhancer of split 4 (TLE4) had interactions between melatonin and exosome biogenesis. It is almost certain that the biogenesis of exosomes was regulated by melatonin pretreatment. Host cells receive external or internal stimuli by inducing specific intracellular signals that then regulate exosome biogenesis (73). The detailed mechanism regarding the effect of melatonin pretreatment on exosome biogenesis still needs to be further clarified in the future.

In addition to regulating exosome biogenesis, we should also consider whether exosomes are qualified to transfer melatonin between cells. Exosomes are a carrier that reveals the nature of the donor cells and the influence of external factors on the donor cells. Direct passive diffusion of melatonin through the cell membrane is acceptable given its lipophilicity and rapid passive diffusion. Researchers continue to explore the mechanism of the transfer of the melatonin receptor MT1 through internalization and endocytic transport (43). When melatonin binds to MT1, the vacuolar sorting machinery uses Rab5 to transfer the internalized MT1 to early endosomes. Endosomes carrying MT1 can cycle to the plasma membrane due to the activity of other GTPases.

## Therapeutic potential of MT-exosomes

Currently, the heterogeneity and mechanism of melatonin pretreatment affecting exosomes are still controversial, but it is undeniable that MT-exosomes have greater therapeutic potential. The use of melatonin pretreatment to enhance the therapeutic effect of exosomes is summarized in **Table 2**.

**Table 2:** Therapeutic effect of MT-exosomes.

Author (Year)	Application	Subjects	Melatonin pretreatment		Therapeutic effect of MT-exosomes		
			Concentration	Time	Anti-inflammatory	Antioxidation	Anti-apoptotic
Yea et al. (2021) (18)	Regulate inflammation and fibrosis	Human AD-derived MSCs/ Male mice	1 μM/ml	24 h	√		√
Heo et al. (2020) (49)	Attenuates inflammation	Human adipose tissue-derived MSCs	10μM	72 h	√		
Wang et al. (2020) (74)	Promote stroke recovery	Male rats			√ TLR4/NF-κB Pathway		
Liu et al. (2020) (15)	Promote diabetic wound healing	hBMSCs and RAW264.7 cell/male rats	1 μmol/L	48 h	√ PTEN/AKT pathway		
Alzahrani et al. (2019) (75)	Treatment of RIRI	BMMSCs/female rats	5 μM	24 h	√	√	√
Sun et al. (2017) (76)	Treatment of acute hepatic ischemia- reperfusion injury.	A macrophage cell line RAW 264.7/male rats	50 µM	3 h	√	√	√
Cheng et al. (2017) (42)	Regulation of immunosuppressive status	Human HCC cell female BALB/c nude mice	0.1 mM		√ STAT3 pathway		

Involve, √; AD, adipose; MSC, mesenchymal stem cells; HCC, hepatocellular carcinoma; RIRI, Renal ischemia-reperfusion injury.

### Anti-inflammatory effects of MT-exosomes

Studies have shown that MT-exosomes significantly improve the polarization of macrophages from proinflammatory macrophages (M1) to anti-inflammatory macrophages (M2) (49). Wang et al. found that melatonin enhances the anti-inflammatory potential of the exosomes through the Toll-like receptors/nuclear factor kappa-B (TLR4/NF-κB) pathway to combat post-stroke inflammation (74). MT-exosomes promote the transformation of macrophages to M2 type by a phosphatase and tensin homolog deleted on the chromosome 10 (PTEN)/AKT signaling pathway (15). MT-exosomes reduce inflammatory cytokines, including interleukin-1β (IL-1β), IL-18, IL-6, and tumor necrosis factor–alpha (TNF-α), increasing the release of anti-inflammatory factors IL-10 and transforming growth factor–β (TGF-β) (**Table 3**).

**Table 3:** Expression of inflammation, oxidation, and expression of apoptotic factors in MT-exosomes.

Author (Year)	Anti-Inflammatory	Inflammatory	Antioxidant status	Oxidative stress status	Anti-apoptotic	Apoptosis	Reference
Yea et al. (2021)		TNF- α↓ NFkB↓				Caspase 3↓	(18)
Heo et al. (2020)	TGF-β↑ IL-10↑ Arg-1↑						(49)
Wang et al. (2020)	TGF-β↑ IL-10↑	TNF-α↓ IL-1β↓ IL-18↓ IL-6↓					(74)
Liu et al. (2020)	IL-10↑ Arg-1↑	TNF-α↓ IL-1β↓ iNOS↓					(15)
Alzahrani et al. (2019)	IL-10↑	NFkB↓ IL-1β↓	HO-1↑ SOD↑ CAT↑ GPx↑	MDA↓ NOX2↓	Bcl2↑	Caspase 3↓ PARP1↓ Bax↓	(75)
Sun et al. (2017)		TNF-α↓ IL-1β↓ MMP-9↓	HO-1↑ NQO1↑	NOX2↓		Caspase 3↓ PARP1↓	(76)
Cheng et al. (2017)		TNF-α↓ IL-1β↓ IL-6↓					(42)

↓, Decrease; ↑, Increase; TGF-β, transforming growth factor–β; IL-10, interleukin-10; Arginine-1; TNF-α, tumor necrosis factor–alpha; IL-1β, interleukin-1β; NFkB, nuclear factor kappa B; IL-18, interleukin-18; MMP-9, matrix metalloproteinase 9; IL-6, interleukin-6; iNOS, inducible nitric oxide synthase; MDA, malondialdehyde; NOX-2, NADPH oxidase 2; HO-1, heme oxygenase-1; NQO1, NAD(P)H quinone dehydrogenase; SOD, superoxide dismutase; CAT, catalase; GPx, glutathione peroxidase; PARP, poly(ADP-ribose) polymerase; Bax, B-cell lymphoma 2–associated X; Bcl2, B-cell lymphoma 2.

**Figure 4:**
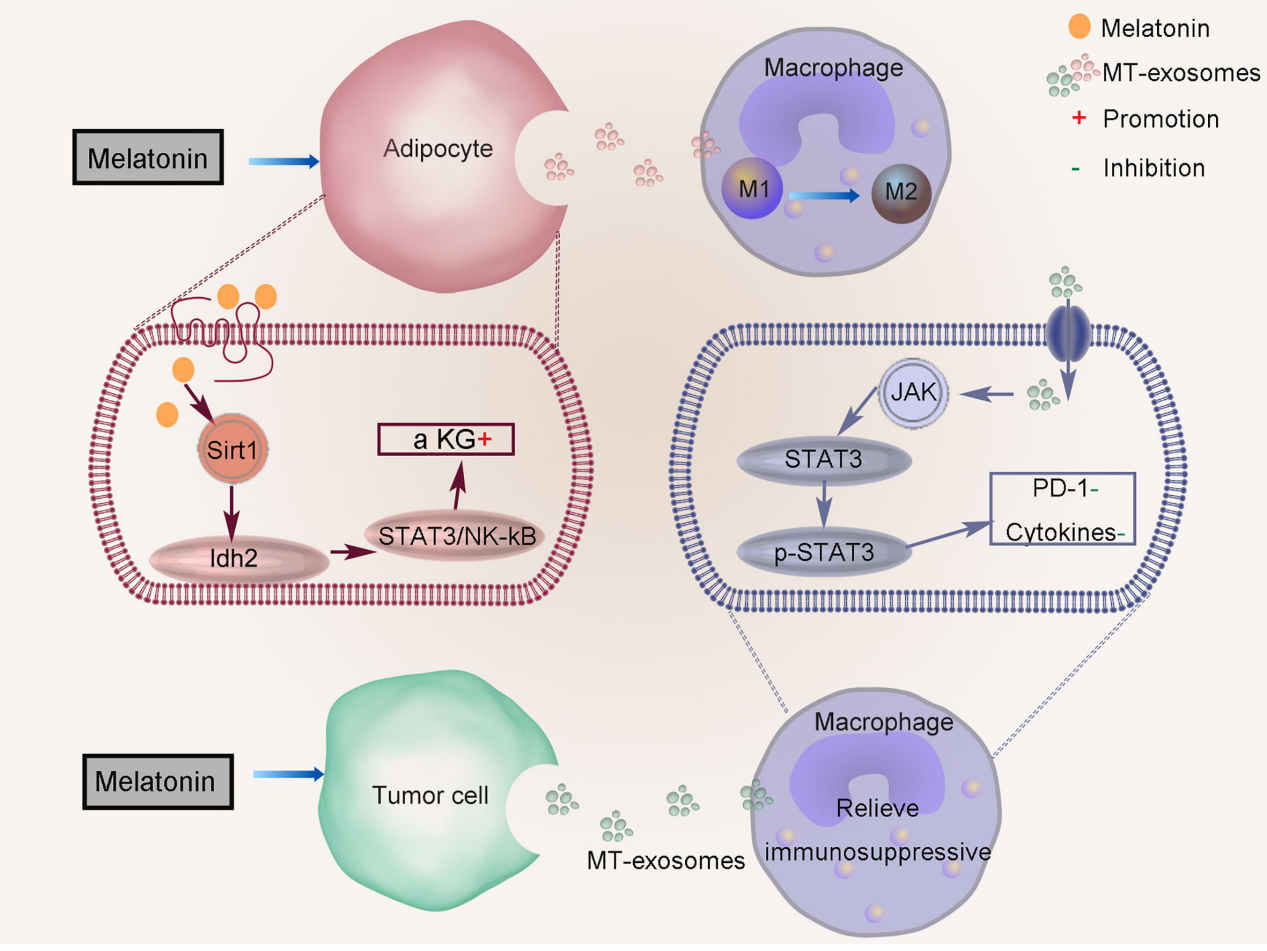
Regulation of anti-inflammatory effect of MT-exosomes. Melatonin mediates the inflammatory response by increasing α-ketoglutarate (α KG) level and transferring to macrophages through exosomes in adipose tissue. MT-exosomes from HCC cells reverse this effect by down-regulating the expression of PD-1 and altering the secretion of macrophage cytokines through inactivating the STAT3 signaling pathway.

Liu et al. suggested that melatonin mediates the inflammatory response by increasing α-ketoglutarate (α-KG) level and transferring to macrophages through exosomes in adipose tissue (**Figure 4**) (53). Melatonin pretreatment promotes the production of α-KG, which increases the DNA demethylation of adipocytes. Melatonin drives the circadian amplitude of mitochondrial isocitrate dehydrogenase 2 (Idh2) in adipose inflammation, and the gene clock regulates Idh2 at the transcription level. Sirtuin 1 (Sirt1) and Idh2 formed a complex to increase α-KG levels that exosomes inactivate signal transducer and the transcriptional activator 3 (STAT3)/NF-kappaB pathways. Exosomes α-KG transports to macrophages, which promote their transformation to M2 and reduce inflammation (TNFα, IL-6, and IL-1β).

The role of MT-exosome is to improve its efficacy based on the therapy of exosomes, which can better alleviate the progression of cell pathology. MT-exosomes can alleviate the pathogenic effects of pathological exosomes on cells, such as exosomes secreted by HCCs. Tumor cells can change the immune status of the tumor microenvironment by upregulating programmed death-ligand 1 (PD-1), and related inflammatory factors such as TNF-a, IL-6, and IL-1β increase significantly. Cheng et al. found that MT-exosomes from HCC cells reverse this effect by downregulating the expression of PD-1 and altering the secretion of macrophage cytokines by inactivating the STAT3 signaling pathway (**Figure 4**) (42).

### Antioxidant and anti-apoptotic effects of MT-exosomes

Melatonin is a powerful antioxidant that scavenges different types of free radicals in body fluids and cells (77, 78). Chen et al. reported that melatonin exerts the protective effect of cellular oxidative stress, also through its anti-apoptosis effect (79). Compared with exosomes, MT-exosomes could play a neuroprotective role by increasing the expression of the anti-apoptotic protein B-cell lymphoma 2 gene (Bcl2) (75).

Inflammation, oxidation, and apoptosis are closely associated with each other. IR injury is a complex, sterile inflammatory cascade (80) and a major cause of cell death and organ damage in many diseases such as myocardial infarction, stroke, and acute kidney injury (81). MT-exosomes have been found to suppress inflammation, oxidative stress, and apoptosis in protecting the liver against IR injury (76). Alzahrani et al. found that treatment with MT-exosomes provided the best protective effect against renal IR injury, compared to the therapy by MSCs or exosomes (75). MT-exosomes decline oxidative stress status (malondialdehyde level and NADPH oxidase 2 protein) and increase antioxidant status (heme oxygenase-1 gene and superoxide dismutase, catalase, glutathione, and peroxidase activities). Moreover, MT-exosomes decline apoptosis (B-cell lymphoma 2–associated X protein genes and caspase 3 activity) and induce an anti-apoptotic effect (Bcl-2) (**Table 3**). Through the expression of antioxidant and anti-apoptotic markers, it is not difficult to find the enhancement of the antioxidant and anti-apoptotic ability of MT-exosomes.

## Usage of melatonin pretreatment

The full use of melatonin pretreatment is firstly summarized in this review. Some studies do not mention whether the concentration of melatonin pretreatment could cause damage to other tissues or any cells. Therefore, we should also pay attention to the potential risks of melatonin pretreatment.

### Route of administration

There are two main methods of pretreatment with melatonin. The former is that melatonin is injected into the body through intravenous injection, and then the blood is collected to extract exosomes (74). The latter is to collect melatonin-pretreated cells and then collect the exosomes secreted by the cells (15).

### Concentration

Based on Amini et al. and Pournagi et al., et al., recent findings about melatonin pretreatment alter exosome size and production in bovine granulosa cells in a dose-dependent manner in which higher-melatonin concentrations (0.1 nM~0.1 mM) contribute to the elevated release of small-sized exosomes (43, 45).

There is no uniform standard for the concentration of melatonin pretreatment *in vivo* and *in vitro* (**Table 2**) (82, 83). Further consideration needs to be given to the variation of optimal concentration of melatonin pretreatment from individual to individual. Through dissolving melatonin in 0.9% saline (with 5% DMSO), Wang et al. injected intraperitoneally (10 mg/kg) the solution for 7 consecutive days and found reduced brain damage in the stroke rats (84).

### Indications

**Figure 5:**
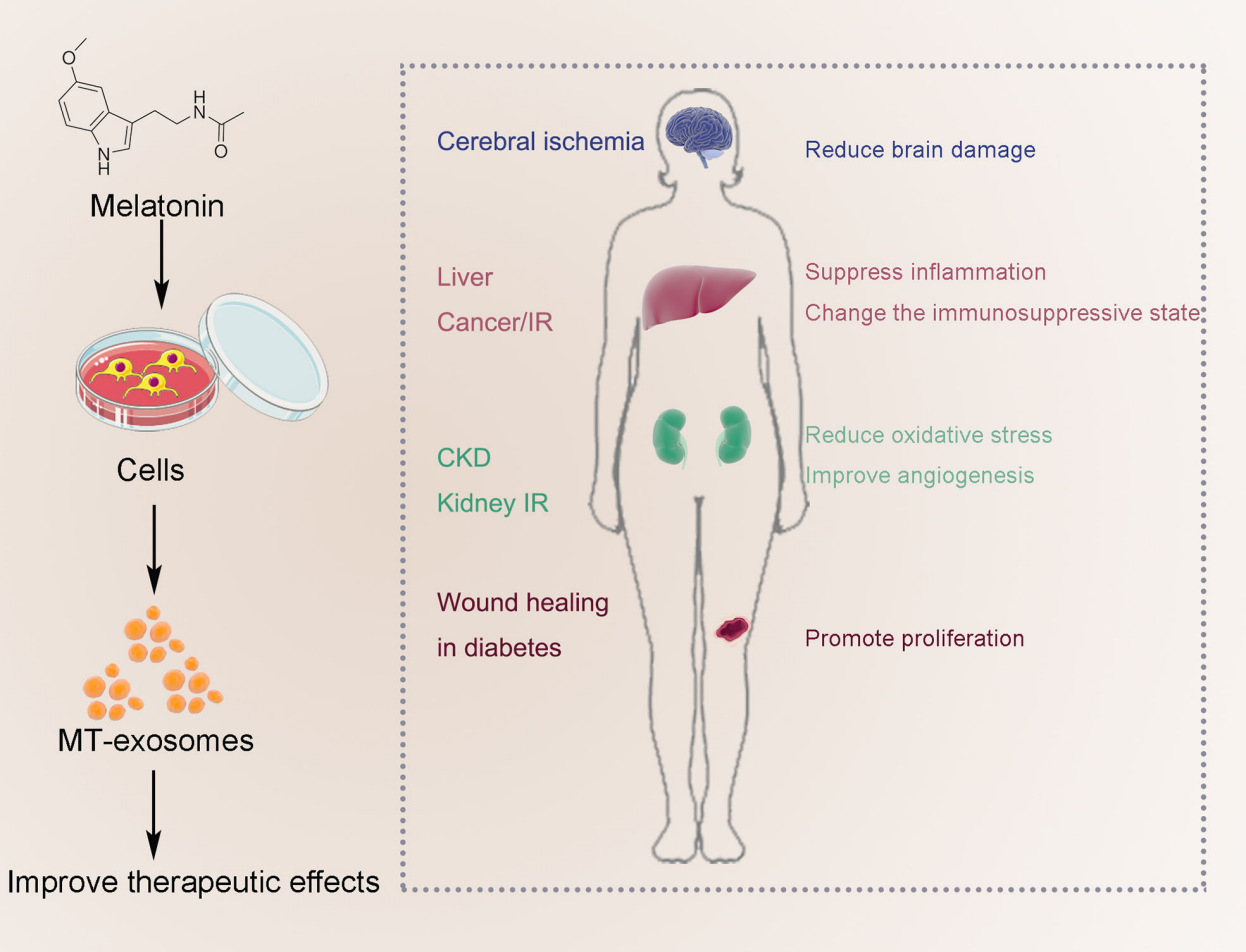
Improve the therapeutic effect of MT-exosomes, mainly including anti-inflammatory and antioxidant effects. Current studies have focused on IR (brain, liver and kidney), liver cancer, AIC, CKD, traumatic spinal cord injury, embryonic development and wound healing in diabetes.

Melatonin can enhance the therapeutic effect of exosomes, mainly including anti-inflammatory and antioxidant effects. Current studies have focused on IR (brain, liver, and kidney), liver cancer, CKD, and wound healing in diabetes (**Figure 5**). As described above, the MT-exosomes were significantly superior to the exosomes, increasing the therapeutic efficiency by about 0.5 to 3 times (42, 74, 75). However, there is currently no unified standard for quantifying the efficiency of exosome treatment. The quantification of exosome therapeutic efficiency should be further confirmed by further studies.

### Potential risks

Melatonin is widely used in medicine and food (24). As observed in animal and human studies, the acute toxicity of melatonin is not lethal even at 800 mg/kg (85). Therefore, the potential risks of melatonin were often overlooked. However, there are different responses to melatonin in clinical trials: mild side effects of melatonin in the short and medium terms, such as irritability, headache, and drowsiness (86, 87). Severe or clinically significant adverse events are rare, including excitement, nightmares, mood swings, fatigue, and skin irritation. Most of these effects either subside spontaneously within a few days without adjusting the dose or subside immediately after discontinuation of the treatment (88, 89). There is no evidence that people develop tolerance to melatonin (85). The risk of melatonin pretreatment has not been clearly reported so far, and we speculate its potential risk through the side effects of melatonin.

Melatonin pretreatment can repair abnormal cells to play its anti-inflammatory and antioxidant role. It is worth considering whether melatonin pretreatment can cause damage to normal cells (90). Melatonin pretreatment is relatively nontoxic but has side effects at high concentrations. The cell survival rate began to decrease when the concentration was higher than 0.1 mM (45). Whether the melatonin pretreatment makes the same effect on the secreted exosomes or not is still unclear. Therefore, we should pay more attention to its potential risks in the future, and more preclinical studies are needed to explore the impact, focusing on possible complications.

## Concluding remarks and perspectives

As nanoscale biological vesicles, exosomes protect their contents from degradation and facilitate their intercellular transfer, and their natural origin and biological properties facilitate the application of exosomes, making them safe and effective for drug delivery. How to better use exosomes as a therapeutic drug has become a focus of attention.

Melatonin is a potent-free radical scavenger and metal chelator with the ability to relieve oxidative stress and inflammatory responses and stabilize cell membranes. Melatonin pretreatment has emerged as a potentially favored alternative approach to enhance exosome function. There are differences in the pretreatment methods of melatonin, including the way, concentration, and time of pretreatment. The melatonin pretreatment can improve the therapeutic potential of exosomes, such as anti-inflammatory, antioxidant, and anti-apoptotic, by changing the proteins and miRNAs carried in exosomes. Related research has focused on cancer, IR, CKD, and wound healing in diabetes. Melatonin is considered one of the most promising approaches to breaking through the dilemma of exosomes.

However, the application of melatonin pretreatment on exosomes is inchoate, and the complexity of exosome heterogeneity makes the exosomes secreted by cells varied after melatonin pretreatment. There are unknown features of the interaction between exosomes with melatonin pretreatment, which needed to be explored in the future: (1) More specific molecular mechanism of melatonin pretreatment on exosomes. We should always remember that mechanisms may vary with specific cell types and conditions. (2) Cross-talk between melatonin pretreatment with different extracellular vesicles subtypes (exosomes are a subtype of extracellular vesicles). (3) The effects of melatonin pretreatment on exosomes from different cell sources and different disease states are likely to be different. (4) Assessing possible risks and application barriers of melatonin pretreatment on exosomes when there is a reproducible effect of melatonin pretreatment on exosomes. (5) Exploring the long-term effects on cells and the exosomes they secrete with melatonin pretreatment, and the possibility of producing exosomes in bulk.

## Author contributions

ZZ and RW contributed equally. ZZ wrote the manuscript. RW was involved in drafting the manuscript. JW, YH, and QX searched the references. XW and LW were responsible for the design and critical revision of the manuscript. All authors read and approved the final manuscript.

## Funding

This work was supported by the National Natural Science Foundation of China (grant numbers 81801004 and 82071155), Shanxi Province Key Research and Development Project (grant numbers 201903D321148), and the Open Project of Shanxi Province Key Laboratory of Oral Diseases Prevention and New Materials (grant numbers KF2020-07).

## Conflict of interest

The authors declare that the research was conducted in the absence of any commercial or financial relationships that could be construed as a potential conflict of interest.

## Publisher’s note

All claims expressed in this article are solely those of the authors and do not necessarily represent those of their affiliated organizations, or those of the publisher, the editors and the reviewers. Any product that may be evaluated in this article, or claim that may be made by its manufacturer, is not guaranteed or endorsed by the publisher.
